# Association of *NOS3*-c.894G>T transversion with susceptibility to metabolic syndrome in Azar-cohort population: A case-control study and *in silico *analysis of the SNP molecular effects 

**DOI:** 10.22038/ijbms.2021.50528.11511

**Published:** 2021-03

**Authors:** Ensiyeh Seyedrezazadeh, Elnaz Faramarzi, Nasim Bakhtiyari, Atefeh Ansarin, Neda Gilani, Amir Amiri-Sadeghan, Maryam Seyyedi, Khalil Ansarin, Younes Aftabi

**Affiliations:** 1 Tuberculosis and Lung Diseases Research Center, Tabriz University of Medical Sciences, Tabriz, Iran; 2 Liver and Gastrointestinal Diseases Research Center, Clinical Research Institute, Tabriz University of Medical Sciences, Tabriz, Iran; 3 Department of Statistics and Epidemiology, Faculty of Health, Tabriz University of Medical Sciences, Tabriz, Iran

**Keywords:** Azar-cohort, Bioinformatics, Metabolic syndrome, Nitric oxide pathway, rs1799983

## Abstract

**Objective(s):**

We investigated whether *NOS3*-c.894G>T transversion (rs1799983), which causes the substitution of glutamate with aspartate (E298D) in the oxygenase domain of endothelial nitric oxide synthase (eNOS), is associated with susceptibility to metabolic syndrome (MetS) risk in Iranian-Azerbaijanis.

**Materials and Methods:**

The frequencies of the alleles and genotypes were compared in the 300 cases and 300 controls using PCR-RFLP assay. Also, higher-order MetS interaction with the genotypes, gender, age, and body mass index (BMI) was evaluated by classification and regression tree (CART) analysis. *In silico* analysis was done to introduce a hypothesis describing the molecular effects of *NOS3*-c.894G>T.

**Results:**

The T allele (OR:1.46; CI:1.054-2.04; *P*=0.02), GT genotype (OR:1.44; CI:1.02-2.03; *P*=0.03), and dominant model (TT+GT vs GG, OR:1.48; CI:1.06-2.06; *P*=0.01) were found to be associated with increased risk of MetS. In the male subpopulation TT genotype (OR:7.19; CI:1.53-33.70; *P*=0.01) was discovered to be associated with increased odds of MetS. CART analysis showed that *NOS3*-c.894G>T genotypes and BMI significantly contribute to modulating MetS risk. Furthermore, *in silico* investigation revealed that c.894G>T may alter eNOS function through affecting interactions of its oxygenase domain with proteins such as B2R, b-actin, CALM1, CAV1, GIT1, HSP90AA1, NOSIP, and NOSTRIN.

**Conclusion:**

We showed that *NOS3*-c.894G>T was associated with an increased risk of MetS in Iranian-Azerbaijanis, and BMI modulates the effects of *NOS3*-c.894G>T genotypes on MetS risk. Also, *in silico* analysis found that *NOS3*-c.894G>T may affect the interaction of the eNOS oxygenase domain with its several functional partners.

## Introduction

Metabolic syndrome (MetS) is defined as a cluster of conditions that occur together more often than by chance alone and increase the risk of cardiovascular diseases and type 2 diabetes (T2D) (1). These conditions include raised blood pressure, raised fasting glucose, dyslipidemia (raised triglycerides and lowered high-density lipoprotein cholesterol), and central obesity (2). Both environmental and genetic factors are widely thought to be contributing to the risk of MetS, and genetic variations have been attributed to genetic predispositions (3). Therefore, investigation of associated genetic variations can improve the current knowledge of MetS etiology.

The nitric oxide (NO) pathway is one of several molecular pathways that contribute to the MetS pathophysiology (4). Although NO is an unstable molecule with a very limited half-life it acts as an effective signaling molecule due to free and fast diffusion through the cytoplasm and cell membranes (5). Three members of the nitric oxide synthase (NOS) family including endothelial NOS (eNOS), inducible NOS, and neuronal NOS are responsible for NO production (6). eNOS is encoded by the *NOS3* gene, which acts as a master regulator of NO synthesis and its physiological level (7). Variations in *NOS3* are major contributors to plasma NO levels and risk of MetS components in different populations (8, 9). 

The *NOS3*-c.894G>T transversion (rsID: rs1799983), which leads to substitution of glutamate (E) code GAG with aspartate (D) code GAT at position 298 (eNOS-E298D) in the oxygenase domain of eNOS (10) is one of the most clinically significant single nucleotide polymorphisms (SNPs) of this gene. The transversion is associated with diabetic nephropathy (11), diabetes mellitus (12), and increased susceptibility to cardiovascular disease (13).

Regarding MetS prevalence in the Iranian population, a meta-analysis was conducted on the 32 studies with a sample size of 83227 cases and found that the total prevalence is 31% (14). Among Iranian people, Azerbaijanis in the northwest of the country have a high prevalence of MetS risk factors such as obesity, serum free fatty acids, hypertension, and high waist circumference (WC) size (15). Due to lack of enough information about the association of *NOS3*-c.894G>T transversion with susceptibility to MetS in Iranians, this study aimed to investigate its association with MetS risk in Iranian-Azerbaijanis in a case-control study. Also, considering that in silico analysis of variations effect may help to further understanding of their molecular outcomes (16) a bioinformatic analysis was conducted to predict the possible consequences of *NOS3*-c.894G>T transversion.

## Materials and Methods

***Study subjects, sample size calculation, and genomic DNA isolation***

Research protocols were approved by the medical ethics committee of the research council of the National Institute for Medical Research Development, Tehran, Iran (IR-NIMAD-REC-1396-032). We calculated the sample size of the study using STATA version 16 and referring to the Stata power and sample-size reference manual (17) and findings of Ianas *et al*. (2009), who studied the association of *NOS3*-c.894G>T with MetS risk in a Romanian sample population (51). Based on this study the “odds ratio (OR) of allele T in cases relative to controls” and “probability of allele T among controls” were considered 1.83 and 0.282, respectively. Also, “Power” and “α” were considered as 0.9 and 0.05, respectively and the order “power mcc 0.282, oratio (1.83) power (0.9)” was executed in the software. The minimum size of the groups was calculated as 255 and considering attrition bias=10% the size raised to 285. We obtained blood samples (collected into sterile tubes containing sodium citrate) and essential data of 300 cases with MetS and 300 healthy controls from the Azar cohort study (15). In this cohort study, subjects were informed about the protocol and purpose of the study and signed a consent form and their data were collected by the questionnaire. Total genomic DNA was isolated from the blood samples by a DNA Extraction Kit (DNAbiotech, Tehran, Iran) following the manufacturer’s instructions. 

***Anthropometric measurements and biochemical parameters determination***

The anthropometric factors including weight, height, and WC were measured and Body Mass Index (BMI) was calculated. Blood pressure was measured twice in one day with an interval of two minutes and twice in each arm in a sitting position after 10 min of rest by a trained nurse using a mercury sphygmomanometer (Rudolf Richter; DE-72417; Germany). The averages of these two measurements were considered as systolic and diastolic blood pressure (SBP and DBP). Blood samples were collected after a 12-hour overnight fast. Fasting blood sugar (FBS), serum triglyceride (TG), and high-density lipoprotein (HDL) were determined by enzymatic methods. Low-density lipoprotein (LDL) was calculated using Friedewald’s formula (18) as modified by Poustchi *et al*. (19). However, considering that the formula is not valuable in subjects with TG≥400 mg/dl, serum LDLs of 18 participants were measured directly.

MetS was defined following criteria reported by the National Cholesterol Education Program (20). Subjects with three or more of the following conditions were defined as MetS cases: WC ≥102 cm in men and ≥88 cm in women, TG>150 mg/dl (drug treatment for elevated triglycerides was an alternate indicator), HDL-C values of <40 mg/dl in men and <50 mg/dl in women. Hypertension was defined as elevated SBP (>130) and/or DBP (>85) mmHg or the use of antihypertensive medication. Elevated fasting glucose was considered to be >100 mg/dl or the use of glucose-lowering medication. 

***Genotyping***

The *NOS3*-c.894G>T transversion was analyzed by the PCR-restriction fragment length polymorphism (PCR-RFLP) method using specific forward 5’-GAAGGCAGGAGACAGTGGAT-3’ and reverse 5’-CAATCCCTTTGGTGCTCACG-3’ primers and restriction enzyme DpnII. PCR was carried out in 20 µl of PCR reaction containing 10 µl Master Mix RED (5200300-1250: Ampliqon, Denmark), 0.5 µl (10 pmol/µl) of each forward and reverse primer (Bioneer, Takapouzist, Iran), and 2 µl of the extracted DNA (50 ngr/µl) as a template in 7.5 µl deionized water. PCR was performed in the Primus thermal cycler (PEQLAB, Erlangen, Germany) with the following conditions: initial denaturation at 94 °C for 5 min, followed by 35 repetitive cycles of denaturation at 94 °C for 45 Sec, annealing at 60 °C for 45 Sec, elongation at 72 °C for 40 Sec, and final elongation at 72 °C for 5 min. 4 µl (about 0.1 µg) of PCR product was digested with 5 units of the DpnII restriction enzyme (BioLabs; UK) and incubated at 37 °C overnight. After digestion, the treated mixture was electrophoresed on 2% agarose gel, and alleles (T: 163+80 bp; G: 243 bp) were visualized by dual intensity transilluminator (UVP, Upland, USA). For quality control, genotyping was done without the knowledge of the control or case status of the subjects. Also, different individuals performed genotyping of a randomly selected 5% of the cases and controls twice. All of the electrophoresis materials were purchased from Fermentas CO., and Merck Co, Germany. Restriction digestions, gel electrophoresis, and staining were performed following the methods described by Green *et al* (21).

***CART analysis***

For the investigation of higher-order interaction between MetS, and genotypes, gender, age, and BMI, classification and regression tree (CART) analysis was performed. It uses nonparametric techniques that examine both linear and nonlinear interactions simultaneously in the whole dataset and creates a hierarchy of association, starting with the strongest to the weakest (22). IBM SPSS Statistics 25.0 (IBM Corp., Armonk, NY, USA) was used to develop models that can classify subjects into MetS risk categories. Several sets of candidate predictors (MetS, genotypes, gender, age, and BMI) were used to build the classification trees. Using several iterations, CART models were used to determine a clinically best logical fit, based on sensitivity and specificity and low estimated risk of the model. The final model included the variables that were potentially related to the risk of MetS. Here, the risk estimate and its standard error for the final model represents a measure of the tree’s predictive accuracy. 

The Gini Index method was used to split off the largest category into a separate group, with the default split size set to enable growing the tree. Gini splits are found that maximize the homogeneity of child nodes concerning the value of the dependent variable. Gini is based on squared probabilities of membership for each category of the dependent variable. The significance level for splitting nodes and merging categories was 0.05. For model estimation, the maximum number of iterations and minimum change in expected cell frequencies were 100 and 0.001, respectively. Also, Pearson chi-square statistics for determining node splitting and category merging were calculated. For multiple comparisons, significance values for merging and splitting criteria are adjusted using the Bonferroni method. When the final tree was built, the tree was pruned, deleting the variables that did not further classify subjects, based on the variable importance score and the sensitivity, into “MetS” or “no MetS” groups. Once a clinically meaningful structure on the CART evolved, pruning was discontinued. Sensitivity, specificity, positive, and negative predictive values were used to assess the performance of the CART model for this sample set. The sensitivity from the CART model was determined using the final “MetS positive” terminal node and specificity was determined using the previous “no MetS” negative terminal nodes. Also, the risk estimate and its standard error were reported as a measure of the tree’s predictive accuracy. For the categorical MetS variable, the risk estimate is the proportion of cases incorrectly classified. 

A set of binomial tests were performed to find whether there are significant differences between the final nodes produced by CART analysis. Also, sensitivity, specificity, positive predictive value (PPV), and negative predictive value (NPV) of the CART analysis were calculated. Sensitivity is the ability of a screening test to detect a true positive and specificity is defined as the ability of a screening test to detect a true negative. PPV refers to the screening test’s power to establish whether people who tested positive on the screening test do or do not actually have the condition of interest. The test’s NPV is the confidence level in a screening test’s ability, when it returns a negative result, to differentiate between people who have a condition and those who do not (23). Sensitivity, specificity, PPV, and NPV are indices of probability and the closer they are to 1 the performance of the associated model is better.

***In silico analysis of the SNP molecular effects***

To find whether *NOS3*-c.894G>T transversion affects eNOS structure and function, we performed sets of in silico analysis. The amino acid sequence of eNOS protein was obtained from UniPort (https://www.uniprot.org) and sequences of eNOS-298E and eNOS-298D variants were prepared. Then, the functional impact of the substitution on the structure and function of eNOS was analyzed using Mutationassessor (24), PhD-SNP (25), PolyPhen- (26), PredictSNP (27), PROVEAN (28), SIFT (29), SNAP (30), and SNAP2 (31) servers. Also, influences of eNOS-E298D in folding characteristics of the protein were analyzed by SNPeffect4 (32), which investigates the effect of a mutation on aggregation propensity (TANGO), amyloid propensity (WALTZ), chaperone binding properties (LIMBO) of protein sequences, and calculates the change of free energy (ddG) after mutation.

Another set of in silico analysis focused specifically on the effects of E298D substitution on the eNOS oxygenase domain. For this purpose at first Pfam (33) was used to identify the sequence of the eNOS oxygenase domain. After that sequences of 298E and 298D variants of the oxygenase domain were subjected to PredictProtein (34) for predicting substitution effects on those features of the domain, which may affect its interaction with other proteins. Finally, proteins that interact or have been predicted to be interacting with eNOS were retrieved from STRING V11 (35) and their interaction with the oxygenase domain was searched in the literature using PubMed and GoogleScholar databases. Furthermore, due to homodimeric nature of active form of eNOS we used the HDOCK Server (36) for docking analysis of eNOS variants: eNOS-298E/eNOS-298E, eNOS-298E/eNOS-298D and eNOS-298D/eNOS-298D.

***Statistical analysis***

IBM SPSS Statistics for Windows (Version 25.0, Armonk, NY: IBM Corp; 2017) software package was used for statistical analysis. Data were presented as mean±SD and frequency (percentage) for the numeric and qualitative variables, respectively. To compare quantitative and qualitative variables between groups, an independent t-test, and chi-square test were used respectively. The distribution of genotypes and alleles was assessed for deviation from the Hardy–Weinberg equilibrium using a chi-square test. Adjusted ORs for the risk of MetS and 95% confidence intervals (CIs) were obtained using logistic regression analyses with adjustments for age and sex. A two-tailed *P*-value of less than 0.05 was considered a significant difference.

## Results

***Study subjects***

As shown in Table 1, there wasn’t any significant difference in gender distribution between the case and control groups. There were significant differences (*P*<0.05) between cases and controls in anthropometric and metabolic factors. Also, gender stratified analysis revealed that for the female subgroup there were significant differences (*P*<0.05) in anthropometric and metabolic factors between cases and controls. Also, in males, except for LDL level, significant differences (*P*<0.05) were observed between cases and controls for the factors.

***Genotyping***

Furthermore, the quality control tests showed that the reproducibility of the obtained results was 100%. Also, transversion did not show significant deviation from the Hardy–Weinberg Equilibrium in the control group. Frequencies of SNP genotypes and alleles in case and control groups were analyzed for the total population (n=600) and stratified male (n=245) and female (n=355) groups.

As shown in Table 2, the frequencies of GT genotype, T allele, and TT+GT vs GG model were significantly associated with increased risk of MetS in the total population after adjustment for age and sex. However, in the male subpopulation, only the TT genotype showed a significant association with increased MetS odds. There was not a significant association between the SNP genotypes and alleles with MetS odds in the female subpopulation after adjusting for age. However, without adjusting GT genotype showed a significant association with increased frequency of MetS (Table 2). Also, there was no significant association between *NOS3*-c.894G>T and MetS components (data not shown).

***CART analysis***

**Figure 1:**
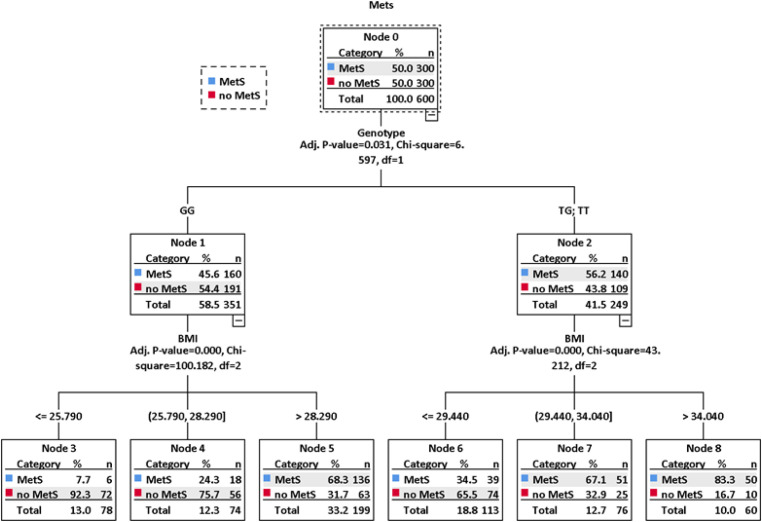
The tree structure of the CART analysis of interaction effects between eNOS-c.894G>T transversion genotypes and BMI in the studied population. Details are discussed in the text

The CART technique creates a decision tree using automatic stepwise variable selection to identify mutually exhaustive and exclusive subgroups of a population. In this study, several sets of candidate predictors (MetS, Genotypes, Sex, Age, and BMI) were used to build the classification trees. Using several iterations, CART models were used to determine a clinically logical fit, based on sensitivity and specificity; the variables included those that were potentially related to the risk of MetS. The cross-validated risk estimate for the final tree was calculated as the average of the risks for all trees and it was 0.07 (Std. error=0.002). When the BMI, age, sex, and genotype were included as independent variables, the number of nodes in the resultant tree structure was nine including six terminal nodes (Figure 1). In the tree there was an initial split on genotypes, confirming that among included factors the genotype is the most important risk factor for MetS. Further inspection of the CART structure suggested distinct patterns for different categories of BMI (Figure 1). 

Binomial tests on the differences of MetS frequency in final nodes of CART analysis (nodes 3, 4, and 5 of GG genotype, and 6, 7, and 8 of GT+TT genotypes) revealed that there was no significant difference (*P*=0.808) in MetS frequency between nodes 3 (BMI≤25.790) and 4 (BMI=25.790-28.290), however, differences between nodes 3 (BMI≤25.790) and 5 (BMI>28.290), and 4 (BMI=25.790-28.290) and 5 (BMI>28.290 were significant (*P*<0.001). In the GT+TT split, the significant differences (*P*<0.001) of MetS frequencies were discovered between nodes 6 (BMI≤29.440) and 7 (BMI=29.440-34.040), and 6 (BMI≤29.440) and 8 (BMI>34.04). However, the difference in MetS frequencies between nodes 7 (BMI=29.440-34.040) and 8 (BMI>34.04) was not significant (*P*=0.198).

Risk estimate is a measure of within-node variance and was used as a criterion of model fit. Lower values indicate a better model. Risk estimate and its standard error were achieved by 0.268 and 0.018, respectively. The algorithm that included genotypes and BMI had sensitivity of 79% (95%CI: 73.95%-83.47%), specificity of 67.33% (95%CI: 61.71%-72.61%), PPV of 70.75% (95%CI: 67.05%-74.19%), and NPV of 76.23% (95%CI: 71.75%-80.19%) for the development sample.

***In silico analysis***

**Figure 2:**
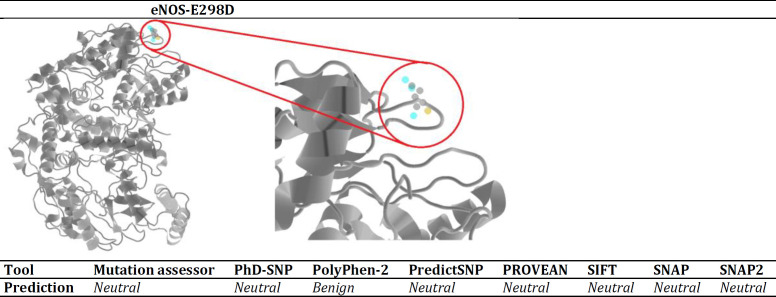
eNOS-E298D position and predicted effect. 3D structure of the eNOS homodimer and E298D position provided by PolyPhen-2 server. Location of the substitution in the surface of protein magnified and depicted in the red circle. The applied servers all reported the effect of substitution as neutral

Based on analysis of position-specific evolutionary preservation and physical and homology properties of residues, the applied servers reported that eNOS-E298D, which occurs on the surface of protein does not affect its structure significantly and therefore it may not result in a structural change-dependent impairment of function (Figure 2).

**Figure 3:**
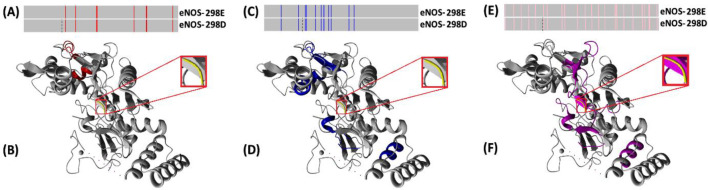
Molecular visualization of SNPeffect4 predictions. In the bar representation, TANGO aggregating stretches are visualized in red, and the dashed vertical line indicates the variant residue (A). Aggregation-prone regions are depicted as red segments in the tertiary structure of eNOS and the location of the variant residue is colored in yellow (B). The position of WALTS amyloid-forming regions in the protein sequence (C) and tertiary structure (D) are visualized in blue. The positions of the LIMBO chaperone-binding sites are depicted in pink in the protein sequence (E) and tertiary structure (F). It is shown that the variant doesn’t affect the responsible sequences for aggregation tendency, amyloid propensity, and chaperone binding tendency

SNPeffect4 calculated dTANGO, dWALTZ, and dLIMBO score for eNOS-E298D was 0.00, which means that it does not affect the aggregation tendency (Figures 3A and B), amyloid propensity (Figures 3C and D), and chaperone binding tendency (Figures 3E and F) of the protein. Also, it reported that the E298D results in a ddG of 0.57 kcal/mol and therefore, slightly reduces the protein stability.

**Figure 4:**
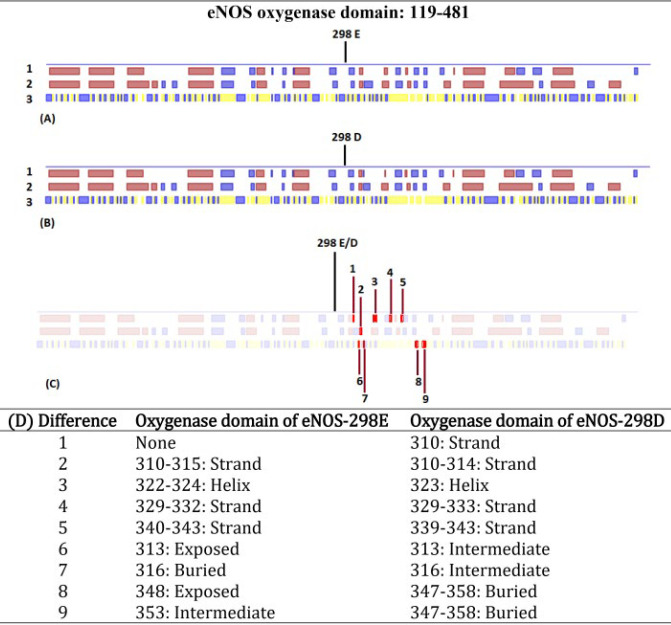
Prediction of secondary structure and solvent accessibility properties of oxygenase domains of eNOS-298E and eNOS-298D by PredictProtein. This viewer lays out predicted features that correspond to regions within the queried sequences of the eNOS oxygenase domain of eNOS-298E (A) and eNOS-298D (B). These panels show results from two prediction methods: RePROFsec, which predicts secondary structure (Line 1) using experimental data, and PROFsec, which predicts secondary structure (Line 2) and solvent accessibility (Line 3) using evolutionary information from multiple sequence alignments and a multi-level system. The pink () and blue () boxes in lines 1 and 2 represent information of local helix and strand elements, respectively. Also, in line 3 the yellow () and blue () boxes depict buried and exposed residues, respectively. In this line, the white-colored sequences represent residues with an intermediate level of exposition. To discover and describe the detailed differences of the predictions for the variants the obtained panels were analyzed using Icy (http://icy.bioimageanalysis.org/), which is an open community platform for bioimage informatics. Nine different parts were identified in the variants (C) and compared (D)

Pfam showed that the sequence 119-481 of eNOS, which contains E298D substitution, makes the oxygenase domain of the protein. Amino acid sequences of the oxygenase domain of eNOS-298E and eNOS-298D were submitted to the PredictProtein and the following results were obtained. PredictProtein aligned submitted sequences with sequences of 76 proteins and analyzed their structure referring to 31 PDB structures. Secondary structure and solvent accessibility properties of the oxygenase domains of eNOS-298E (Figure 4A) and eNOS-298D (Figure 4B) were described by the tool. There were some differences in these properties between the two variants (Figures 4C and D). Helix and strand pattern of the local sequence were changed in the region and there were different exposition features for adjacent residues of the substitution. 

**Figure 5:**
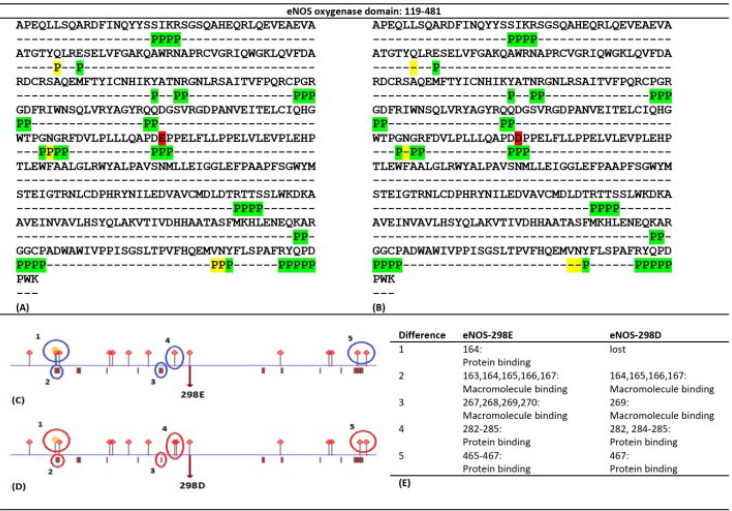
Interaction-involved sequences of oxygenase domains of eNOS-298E and eNOS-298D predicted by PredictProtein. PredictProtein identified residues involved in external PPIs in the oxygenase domain of eNOS-298E (A) and eNOS-298D (B). Residues 298E and 298D are marked by red color. The same and different sites of the two variants are highlighted in green and yellow, respectively. As depicted in the figure the variation may affect the pattern of PPI sites in its up-and-down-stream sequences. “P” stands for “Predicted”. Predicted protein- and macromolecule-binding sites of the oxygenase domain of eNOS-298E (C) and eNOS-298D (D) by the ProNA2019 algorithm. Differences of variants in protein- and macromolecule-binding sites are listed (E)

**Figure 6:**
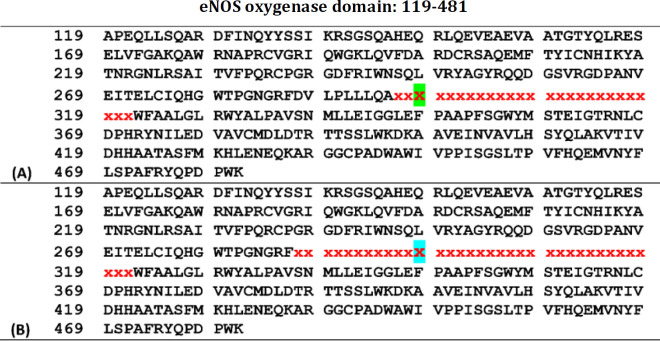
Prediction of low complexity segments in the oxygenase domain of eNOS-298E and eNOS-298D by PredictProtein. PredictProtein found low complexity segments in eNOS-298E (A) and eNOS-298D (B) using the SEG algorithm. The residue X highlighted green in the upper sequence and X highlighted blue in the lower sequence corresponds to amino acids 298E and 298D, respectively. The figure depicts that oxygenase domains of eNOS-298E and eNOS-298D have different low-complexity segments

Furthermore, PredictProtein identified residues involved in external protein-protein interactions (PPIs) in the oxygenase domain of eNOS-298E (Figure 5A) and eNOS-298D (Figure 5B). E298D substitution may affect the pattern of PPI sites in the oxygenase domain of the variants. Also, PredictProtein detected protein- and macromolecule-binding sites of the oxygenase domain of eNOS-298E (Figure 5C) and eNOS-298D (Figure 5D). This set of analyses revealed that oxygenase domains of eNOS-298E and eNOS-298D represent different distribution patterns of involved residues in PPIs and different sites for protein and macromolecule bindings (Figure 5E). Also, PredictProtein used the SEG program to identify low-complexity segments in the variants and revealed that E298D substitution resulted in changing local compositional complexity of oxygenase domain of eNOS-298E (Figure 6A) and eNOS-298D (Figure 6B).

**Figure 7:**
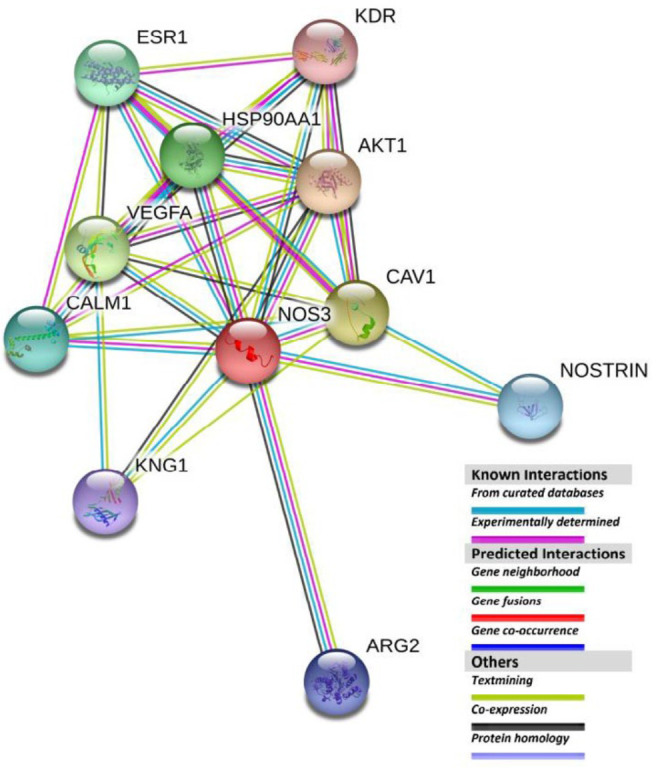
Functional partners of eNOS reported by STRING V11. AKT1 is a serine/threonine- protein kinase, which regulates many processes including metabolism, proliferation, cell survival, growth, and angiogenesis. Caveolin-1 (CAV1) acts as a scaffolding protein within caveolar membranes and interacts directly with G-protein alpha subunits and regulates their activity. Vascular endothelial growth factor A (VEGFA) induces endothelial cell proliferation, promotes cell migration, inhibits apoptosis, and induces permeabilization of blood vessels. Heat shock protein HSP 90-alpha (HSP90AA1) is a molecular chaperone with ATPase activity that promotes the maturation, structural maintenance, and proper regulation of specific client proteins. Estrogen receptor (ESR1) influences nuclear transactivation and affects cellular proliferation and differentiation by binding to estrogen response elements. Calmodulin-1 (CALM1) mediates the control of a large number of enzymes, ion channels, aquaporins, and other proteins through Ca2+-binding. NOSTRIN contains special domains and motifs that regulate eNOS trafficking to membranes. Mitochondrial Arginase-2 (ARG2) regulates extra-urea cycle arginine metabolism and down-regulates NO synthesis. Kininogen-1 (KNG1) plays an important role in many pathophysiological processes including fibrinolysis, thrombosis, inflammation, and oncogenesis. Vascular endothelial growth factor receptor 2 (KDR) is a tyrosine-protein kinase that acts as a cell-surface receptor for VEGFA, VEGFC, and VEGFD and plays an essential role in the regulation of angiogenesis, vascular development, vascular permeability, and embryonic hematopoiesis. In all these cases association does not necessarily mean physically binding. However, by reviewing the literature it is concluded that among STRING-reported functional partners of eNOS, CAV1 (37), HSP90AA1 (38), CALM1 (39), and NOSTRIN (40) interact directly with the eNOS oxygenase domain

STRING V11 was used to find the functional partners of eNOS. As depicted in Figure 7 it identified a core list of eNOS-interacting proteins including Protein kinase B (AKT1), Caveolin-1 (CAV1), Vascular endothelial growth factor A (VEGFA), Heat shock protein 90 alpha family class A member 1 (HSP90AA1), Estrogen Receptor 1 (ESR1), Calmodulin 1 (CALM1), Endothelial nitric oxide synthase traffic inducer (NOSTRIN), Arginase type II (ARG2), Kininogen 1 (KNG1), and Kinase Insert Domain Receptor (KDR). Afterward, searching PubMed and Google Scholar databases for literature on the interacting partners of the eNOS-oxygenase domain we found that among STRING reported partners, CAV1 (37), HSP90AA1 (38), CALM1 (39), and NOSTRIN (40) have direct interaction with this domain. Furthermore, the literature review revealed that there are other eNOS interacting proteins out of STRING identified core list that bind to the protein via its oxygenase domain: Bradykinin Receptor B2 (B2R), which is a G protein-coupled cell surface receptor (41), G-protein-coupled receptor kinase-interacting protein 1 (GIT1) (42), Nitric oxide synthase-interacting protein (NOSIP) (43), and b-actin (44).

**Figure 8:**
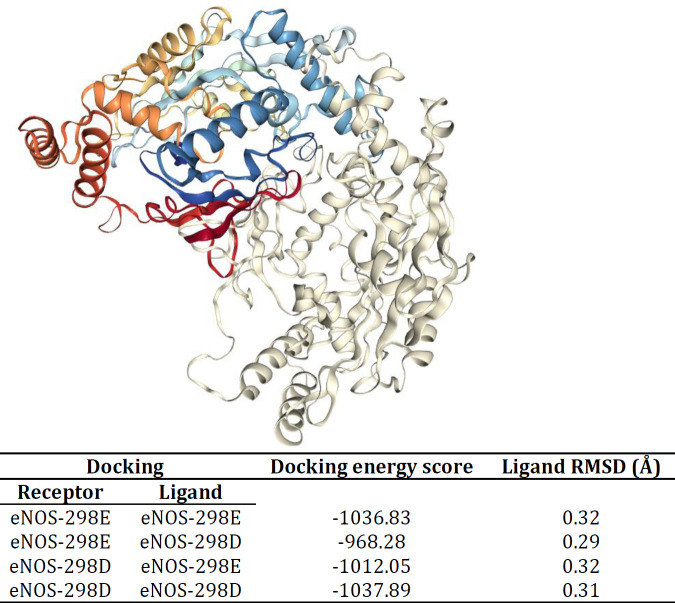
Dimerization of eNOS-298E and eNOS-298D variants using HDOCK Server. As reported by HDOCK Server, there are minor differences between the docking energy scores of eNOS-298E/eNOS-298E and eNOS-298D/eNOS-298D homodimeric structures. However, when the dimer structure was made of eNOS-298E and eNOS-298D docking energy scores were slightly different

As reported by HDOCK Server (Figure 8), there are minor differences between the docking energy scores of homodimeric structures of eNOS-298E and eNOS-298D variants. However, docking energy scores for the mixed dimer of eNOS-298E and eNOS-298D variants were different. Also, RMSD (Root mean square deviation) of eNOS-298E variant in both eNOS-298E/eNOS-298E and eNOS-298D/eNOS-298E dimers was the same (0.32). However, the eNOS-298D variant had slightly changed RMSD when it submitted as a ligand in eNOS-298E/eNOS-298D (RMSD=0.29) or eNOS-298D/eNOS-298D dimer (RMSD=0.31). Considering that G and T alleles of *NOS3*-c.894G>T are responsible for E and D amino acids at the position 298 of eNOS, therefore, the homodimeric structure of eNOS by GT genotype may represent different characteristics (Figure 8).

**Table 1:** MetS components and anthropometric factors of the participants

**Parameters**	**MetS patients (n=300)**	**Controls (n=300)**	***P*** **-value **
**Total**	mean **± **SD	mean **± **SD	
Age (years)	42.97±4.42	41.71±4.15	*0.001
Male n(%)	116(38.7)	129(43)	**0.31
Female n(%)	184(61.3)	171(57)	
Weight (kg)	84.80±13.22	74.11±13.17	*<0.001
BMI (kg/m2)	32.07±4.14	27.71±4.94	*<0.001
WC (cm)	99.25±9.27	87.85±10.24	*<0.001
SBP (mmHg)	121.40±14.91	112.37±12.47	*<0.001
DBP (mmHg)	79.95±8.76	73.07±8.19	*<0.001
FBS (mg/dL)	99.38±13.21	91.29±9.42	*<0.001
Total cholesterol (mg/dL)	204.98±43.93	188.90±33.58	*<0.001
LDL (mg/dL)	121.70±38.3	115.01±30.95	*0.01
HDL (mg/dL)	40.53±9.81	49.56±11.74	*<0.001
Triglycerides (mg/dL)	215.27±123.40	121.5344.41	*<0.001

*P independent t-test; **P chi-square test

**Table 2:** Analysis of NOS3 c.894G>T association with MetS

**Genotype/Allele**	**MetS patients** **N (%)**	**Controls** **N (%) **	**Unadjusted odds ratio (95% CI)**	***P***	*Adjusted odds ratio (95% CI)	***P***
**Total (600)**	300 (50)	300 (50)				
**GG**	160(53.3)	191(63.7)	1(ref.)			
**GT**	122(40.7)	98(32.7)	1.48(1.05-2.08)	0.02	1.44(1.02-2.03)	**0.03**
**TT**	18(6)	11(3.7)	1.95(0.89-4.25)	0.09	1.84(0.84-4.06)	0.12
**G**	442(73.7)	480(80)	1(ref.)			
**T**	158(26.3)	120(20)	1.51(1.09-2.09)	0.01	1.46(1.054-2.04)	**0.02**
**GT+TT vs GG**	140(46.7) vs 160(53.3)	109(36.3) vs 191(63.7)	1.53(1.10-2.12)	0.01	1.48(1.06-2.06)	**0.01**
HWE**	Chi^2 ^= 0.7; p>0.05	Chi^2 ^= 0.13; p>0.05				
**Male (n=245)**	116	129				
**GG**	63(54.3)	84(65.1)	1(ref.)			
**GT**	42(36.2)	43(33.3)	1.3(0.76-2.22)	0.33	1.30(0.76-2.23)	0.33
**TT**	11(9.5)	2(1.6)	7.33(1.5-34.26)	0.01	7.19(1.53-33.70)	**0.01**
**G**	179(77.2)	213(82.6)	1(ref.)			
**T**	53(22.8)	45(17.4)	1.57(0.93-2.62)	0.08	1.56(0.93-2.62)	0.08
**GT+TT vs GG**	53(45.7) vs 63(54.3)	45(34.9) vs 84(65.1)	1.57(0.93-2.62)	0.08	1.56(0.98-2.29)	0.08
**HWE**	Chi^2 ^= 1.02; p>0.05	Chi^2 ^=1.82; p>0.05				
**Female (n=355)**	184	171				
**GG**	97(52.7)	107(62.6)	1(ref.)			
**GT**	80(43.5)	55(32.2)	1.60(1.03-2.49)	0.03	1.50(0.96-2.35)	**0.07**
**TT**	7(3.8)	9(5.3)	0.85(0.30-2.39)	0.77	0.78(0.27-2.23)	0.65
**G**	263(71.5)	267(78.1)	1(ref.)			
**T**	105(28.5)	75(21.9)	1.46(0.96-2.24)	0.07	1.37(0.89-2.11)	0.15
**GT+TT vs GG**	87(47.3) vs 97(52.7)	64(37.4) vs 107(62.6)	1.5(0.98-2.29)	0.06	1.4(0.91-2.16)	0.12
**HWE**	Chi^2 ^= 3.76; p>0.05	Chi^2 ^= 0.3; p>0.05				

*Adjusted odds ratio (95% CI) for age and sex in the total sample of study; adjusted for age in genders

** Hardy–Weinberg Equilibrium

## Discussion

Here we showed that the transversion *NOS3*-c.894G>T is associated with an increased risk of MetS among the studied population. The T allele, heterozygote genotype GT, and TT+GT vs GG dominant model were associated with increased risk of MetS in the total population. Also, in the male subpopulation, homozygote TT genotype was associated with an increased risk of MetS. However, in the female subpopulation, there wasn’t a significant association except for the GT genotype, which was associated with increased risk of MetS, but it didn’t remain significant after adjustment for age. Furthermore, CART analysis classified individuals into subgroups with different BMI profiles based on their distinct genotype backgrounds. It showed a significant interaction between BMI, the SNP genotypes, and MetS risk in the population. 

This polymorphism has been previously investigated in Iranian populations in the context of its association with coronary artery disease and T2D, which are the main consequences of MetS (45, 46). Mahmoodi *et al*. found that in a sample population of Zanjan, northwest Iran, *NOS3*-c.894G>T is a significant risk factor for the development of coronary artery disease via reducing the plasma levels of NO (47). Also, a study reported an association of SNP with coronary artery disease for the population of Kermanshah in western Iran (46). 

MetS includes dyslipidemia, hyperglycemia, hypertension, diabetes, and obesity, and there is an association between *NOS3* gene polymorphisms and these features (48). For instance, in the Slavic population, patients with MetS carrying TT genotype of the *NOS3*-c.894G>T transversion had higher serum endothelin-1 (ET-1) levels in comparison with GT and GG carriers (49), and higher serum ET-1 is reported to be associated with MetS risk (50). Ianas *et al*. reported that in the Romanian people T allele and GT and TT genotypes of *NOS3*-c.894G>T polymorphism were associated with susceptibility to the endocrine changes involved in the pathogenesis of MetS (51).

E298D substitution lies within the oxygenase domain of eNOS, where the substrate L-arginine is bound (52) and oxidized into the intermediate OH-L-arginine, which is then oxidized into L-citrulline and NO (53). The affinity changes of this bounding could alter eNOS pathway outputs and affect key processes such as vasodilatation, antithrombotic action, blood pressure, and vascular homeostasis (54). Although there are some opposed reports (55), it is previously demonstrated that the D variant leads to a reduced production level of NO in *in vivo *cases (47) and also twofold less enzymatic activity in the human placenta has been detected (56). It is noteworthy that a study reported a direct association between the metabolic risk factors and the concentration of serum NO and showed that it was significantly higher in subjects with MetS (57). However, another study reported that the increase of NO concentration did not depend on the presence of GG and GT genotypes in the Russian population (58). 

In the current study, the effects of eNOS-E298D on eNOS structure and function were tested using bioinformatics approaches. The capabilities of bioinformatics analysis in SNP studies (59–61) including SNPs of *NOS3* (62) have been shown previously. Direct interaction of the oxygenase or reductase domains of eNOS with the functional partners play an important role in the regulation of protein activities (63). PredictProtein disclosed that the substitution can affect the secondary structure and solvent accessibility properties of the domain by changing helix and strand pattern of the local sequence in the region and altering exposition features for adjacent residues of eNOS-E298D. Furthermore, it showed that low-complexity segments in the variants of the oxygenase domain were changed due to eNOS-E298D substitution. Low-complexity regions in proteins are sequences with biased amino acid composition, which show remarkable degrees of compositional plasticity. These regions may be involved in flexible binding associated with specific functions (64). Therefore, with these changes, eNOS-E298D may affect the interactions of the eNOS-oxygenase domain. 

To find and specify whether this domain interacts with especial partners we used the STRING tool and surveyed the related literature. STRING identified 11 eNOS-interacting proteins, among which literature search showed, four proteins CAV1 (37), HSP90AA1 (38), CALM1 (39), and NOSTRIN (40) bound to eNOS through its oxygenase domain. However, other discovered interactions have been reported for eNOS. Review of literature showed that, in addition to STRING-identified oxygenase-domain interacting partners, proteins such as B2R (41), b-actin (44), GIT1 (42), and NOSIP (43) also bind to the eNOS-oxygenase domain. These interactions show the importance of the eNOS-oxygenase domain in trafficking, localization, enzymatic activity, and regulation of eNOS function. Researchers showed that in E/E and D/D mutant genotypes, the amount of eNOS associated with CAV1 was significantly lower and predicted that E298D might affect the localization of eNOS (65). Furthermore, changing the dimerization features of eNOS may be another target of E298D substitution, which can affect producing the functional homodimeric structure of the enzyme. Therefore, theoretically, E298D may affect a broad range of oxygenase domain interactions and enzyme dimerization, however, more detailed molecular studies are needed to discover the effects and their cellular consequences. In this study lacking participant NO data was the main limitation and also, we could not analyze the possible gene-environment and gene-gene interactions that may affect the impact of the studied SNP.

## Conclusion

This case-control study revealed that *NOS3*-c.894G>T transversion is associated with metabolic syndrome risk among Iranian-Azerbaijanis. Also, CART analysis found the importance of BMI in modulating of *NOS3*-c.894G>T effect on MetS risk. Finally, our *in silico* analysis suggested that *NOS3*-c.894G>T may impose influences on the interactions of the oxygenase domain of eNOS protein with several functional partners and affect the overall activity of the enzyme through changing the protein PPI, however, the molecular and cellular results of these alterations and their effect on eNOS activity and regulation demand more meticulous investigations.
